# Supplementary material for: Microtubule end stabilisation by cooperative oligomers of Ska and Ndc80 complexes
Source: EMBO J. 2026 Mar 20;45(9):2905–37. doi: 10.1038/s44318-026-00749-5 (PMC13144512; doi:10.1038/s44318-026-00749-5)

Appendix for

Microtubule end stabilisation by cooperative oligomers of Ska and Ndc80 complexes

Renjith M. Radhakrishnan[1], Lauren Stokes[1], Matthew Day[1], Pim J. Huis in 't Veld[2,3,4], Vladimir A. Volkov[1]*

1. Centre for Molecular Cell Biology, School of Biological and Behavioural Sciences, Queen Mary University of London, United Kingdom;
2. Department of Mechanistic Cell Biology, Max Planck Institute of Molecular Physiology, Dortmund, Germany.
3. Max Perutz Labs, Vienna BioCenter (VBC), Vienna, Austria
4. University of Vienna, Max Perutz Labs, Vienna, Austria.

Table of contens:

Appendix Figure S1. (A) Representative immunofluorescence confocal images of cold-treated cells with poor expression of GFP-SKA1 wt, following siRNA of endogenous SKA1. Individual confocal planes are shown. Magenta – ACA, yellow – GFP-SKA1, cyan – tubulin. (B) Representative immunofluorescence confocal images of cold-treated cells expressing GFP-SKA1 R236A with normal chromosome alignment. (C) Representative immunofluorescence confocal images of cold-treated cells expressing GFP-SKA1 R236A with misaligned chromosomes. Scale bars: 5 μm.

Appendix Figure S1

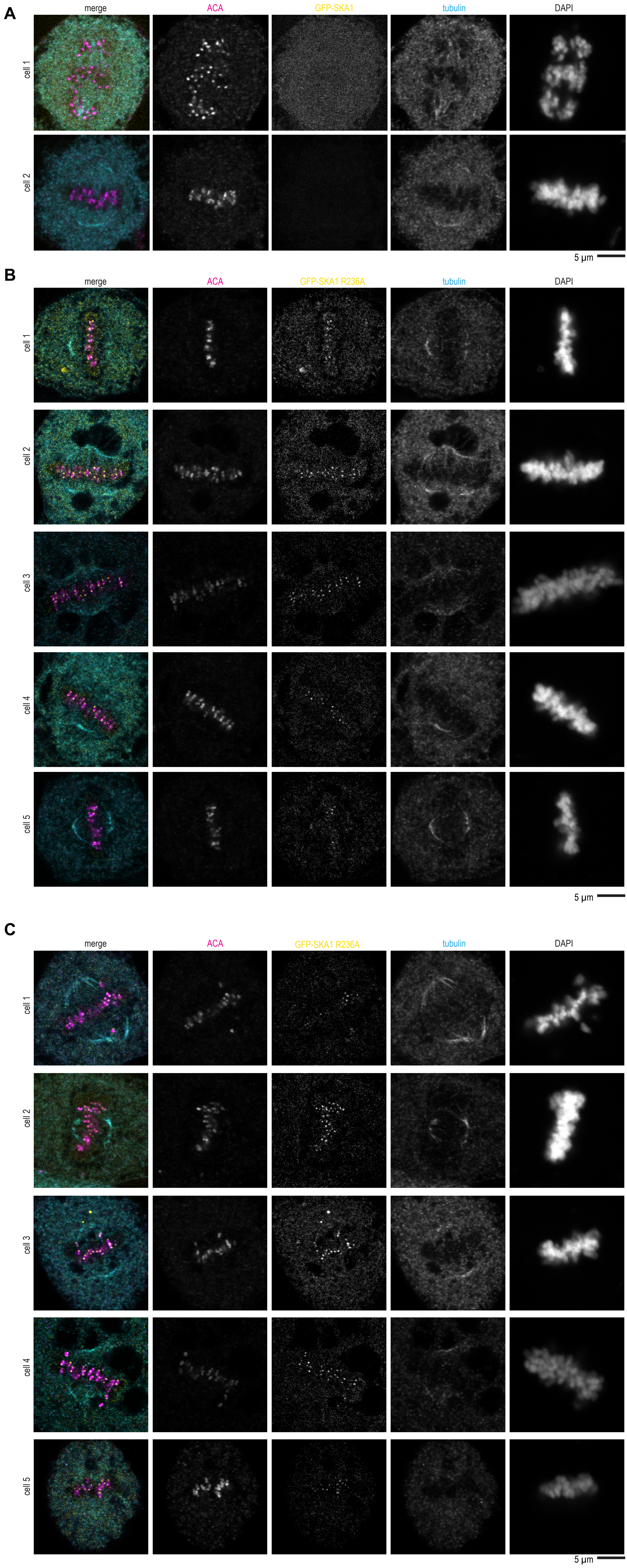

Supplement: Supplementary file 1 — Appendix [file 44318_2026_749_MOESM1_ESM.pdf]
