# Supplementary figures and images for: Microtubule end stabilisation by cooperative oligomers of Ska and Ndc80 complexes
Source: EMBO J. 2026 Mar 20;45(9):2905–37. doi: 10.1038/s44318-026-00749-5 (PMC13144512; doi:10.1038/s44318-026-00749-5)

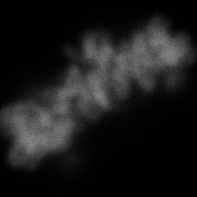

Supplement: Supplementary file 12 — Source data Fig. 7 [file 44318_2026_749_MOESM12_ESM.zip › Fig7/7C/R236A_DAPI.tif]
