# Supplementary material for: Microtubule end stabilisation by cooperative oligomers of Ska and Ndc80 complexes
Source: EMBO J. 2026 Mar 20;45(9):2905–37. doi: 10.1038/s44318-026-00749-5 (PMC13144512; doi:10.1038/s44318-026-00749-5)
Supplement: Supplementary file 14 — Expanded View Figures [file 44318_2026_749_MOESM14_ESM.pdf]

Expanded View Figures

Figure EV1. Purification of Ndc80 and Ska complexes, and Cdk1 phosphorylation of Ska.

(**A**) SDS-PAGE of full-length Ndc80 and Ska complexes, using Coomassie staining (top), and in-gel fluorescence of TMR (left) or AzDye-488 (right). (**B**) SDS-PAGE of the CyclinB/Cdk1/ CKS1 complex (left) and Ska complex treated with CyclinB/Cdk1/CKS1 next to an untreated control, and Ska$^{SKA3\ T358/360A}$, Ska$^{SKA3\ \Delta351\text{-}377}$, and Ska$^{SKA3\Delta C}$. (**C**) Comparison of the microtubule-stabilising activity of Cdk1-treated and untreated Ska, and Ska$^{SKA3\ T358/360A}$ in presence of an indicated concentration of Ndc80 (mean ± SD). Two-way ANOVA comparing hyperphosphorylated and untreated Ska: row factor (Ndc80 concentration) $p = 0.0003$ (**); column factor (Ska treatment) $p = 0.33$ (n.s.). Two-way ANOVA comparing Ska$^{SKA3\ wt}$ and Ska$^{SKA3\ T358/360A}$: row factor (Ndc80 concentration) $p = 0.0092$ (**); column factor (Ska wt vs mutant) $p = 0.0015$ (**). At least 50 seeds quantified in total over at least 5 fields of view per repeat, repeated 2–3 times. (**D**) Length of residual fluorescent microtubule extensions after tubulin dilution in presence of the proteins in concentrations indicated. Dots: individual measurements, lines: mean ± SD. (**E**) Microtubule decoration by Ndc80 and Cdk1-treated and untreated Ska. Scale bar: 5 µm.

© The Author(s)

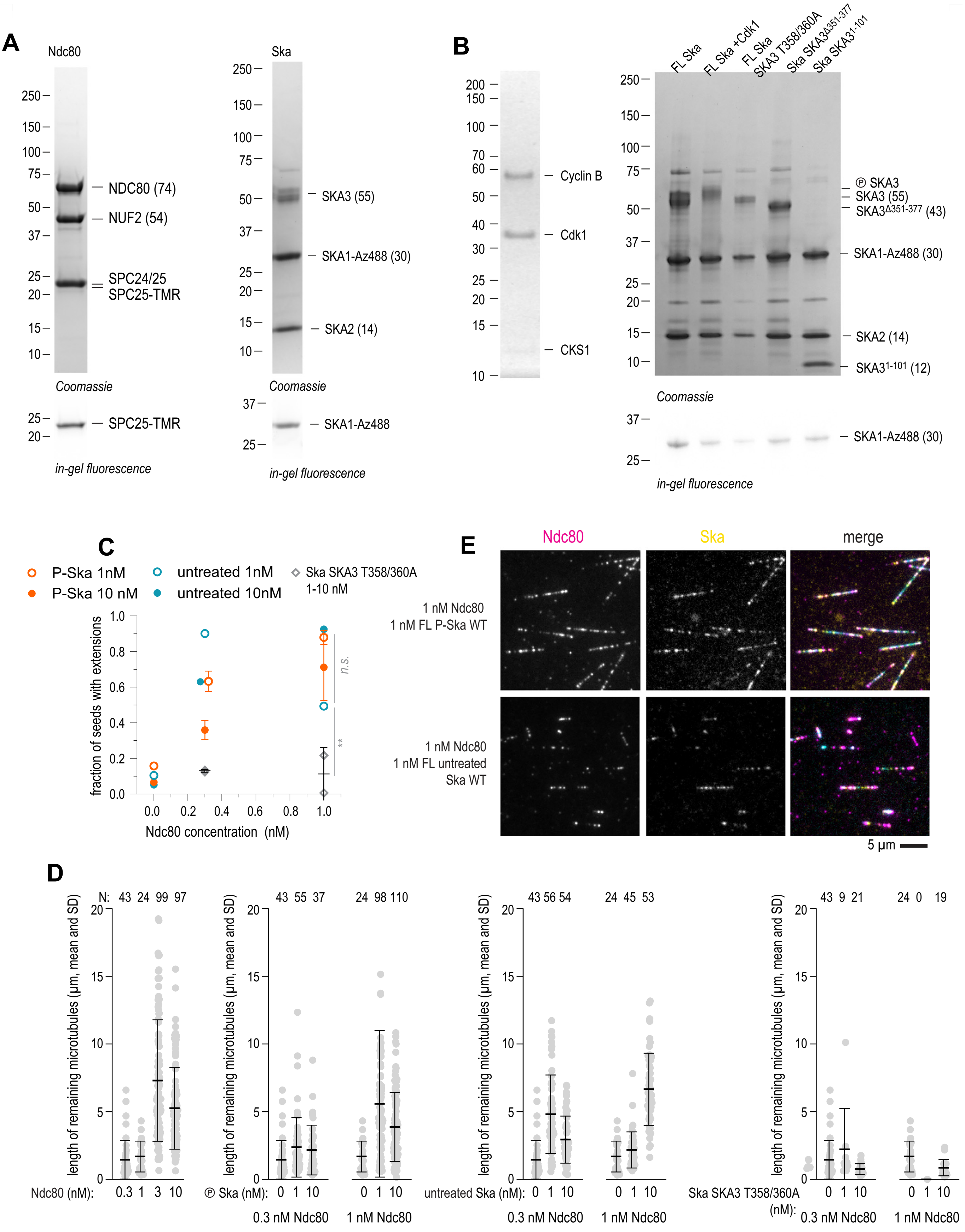
A
Ndc80
NDC80 (74)
NUF2 (54)
SPC24/25
SPC25-TMR
Coomassie
SPC25-TMR
in-gel fluorescence
Ska
SKA3 (55)
SKA1-Az488 (30)
SKA2 (14)
Coomassie
SKA1-Az488
in-gel fluorescence
B
Cyclin B
Cdk1
CKS1
FL Ska
FL Ska +Cdk1
FL Ska SKA3 T358/360A
Ska SKA3Δ351-377
Ska SKA31-101
Ⓟ SKA3
SKA3 (55)
SKA3Δ351-377 (43)
SKA1-Az488 (30)
SKA2 (14)
SKA31-101 (12)
Coomassie
SKA1-Az488 (30)
in-gel fluorescence
C
P-Ska 1nM
P-Ska 10 nM
untreated 1nM
untreated 10nM
Ska SKA3 T358/360A 1-10 nM
fraction of seeds with extensions
Ndc80 concentration (nM)
n.s.
**
E
Ndc80
Ska
merge
1 nM Ndc80
1 nM FL P-Ska WT
1 nM Ndc80
1 nM FL untreated Ska WT
5 µm
D
length of remaining microtubules (µm, mean and SD)
N: 43 24 99 97
Ndc80 (nM): 0.3 1 3 10
43 55 37
24 98 110
Ⓟ Ska (nM): 0 1 10
0.3 nM Ndc80
1 nM Ndc80
43 56 54
24 45 53
untreated Ska (nM): 0 1 10
0.3 nM Ndc80
1 nM Ndc80
43 9 21
24 0 19
Ska SKA3 T358/360A (nM): 0 1 10
0.3 nM Ndc80
1 nM Ndc80

 © The Author(s)

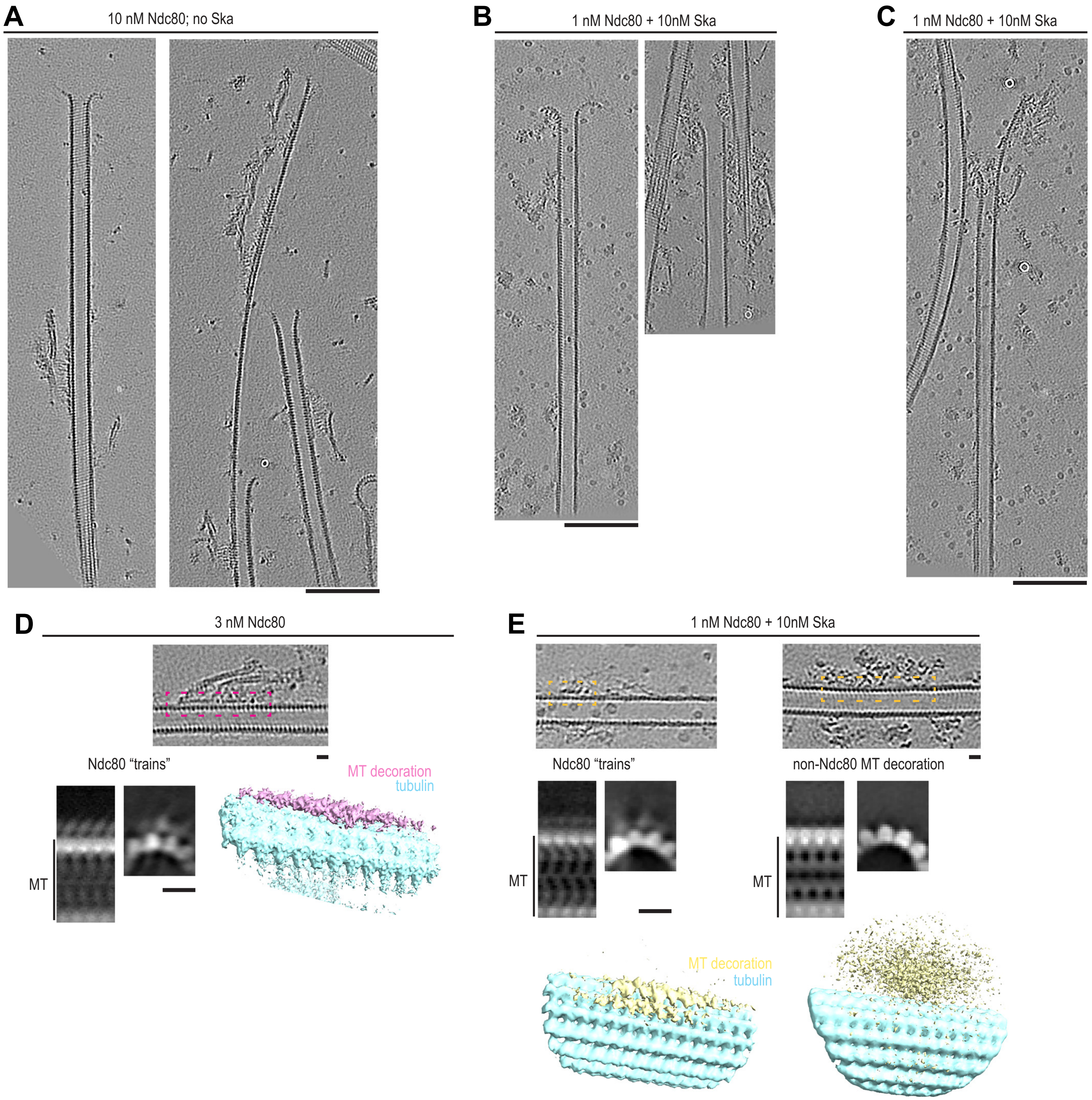

Figure EV2. Characterisation of Ndc80 and Ska oligomers on microtubules using cryoET.

Additional examples of microtubules decorated with Ndc80 trains at 10 nM Ndc80 (**A**), with non-Ndc80 oligomers in presence of 1 nM Ndc80 and 10 nM Ska (**B**), and with Ndc80 trains stabilising extended sheet-like protofilaments in presence of 1 nM Ndc80 and 10 nM Ska (**C**). (**D**) Subtomogram average of Ndc80 trains in a sample with 3 nM Ndc80: 1285 particles obtained from 26 tomograms, deposited to EMBD as EMD-56086. Images shown are binned by 2 compared to the deposited map. (**E**) Subtomogram averaging of CH-domain trains (left), and non-Ndc80 microtubule decorations in the sample containing 1 nM Ndc80 and 10 nM Ska. White-on-black images represent 2D projections of 3D classes. Colour images show 3D rendering of the same classes. Ndc80 "trains": 822 particles obtained from 42 tomograms (EMD-56088). Non-Ndc80 densities: 5026 particles from the same 42 tomograms (EMD-56087).

© The Author(s)

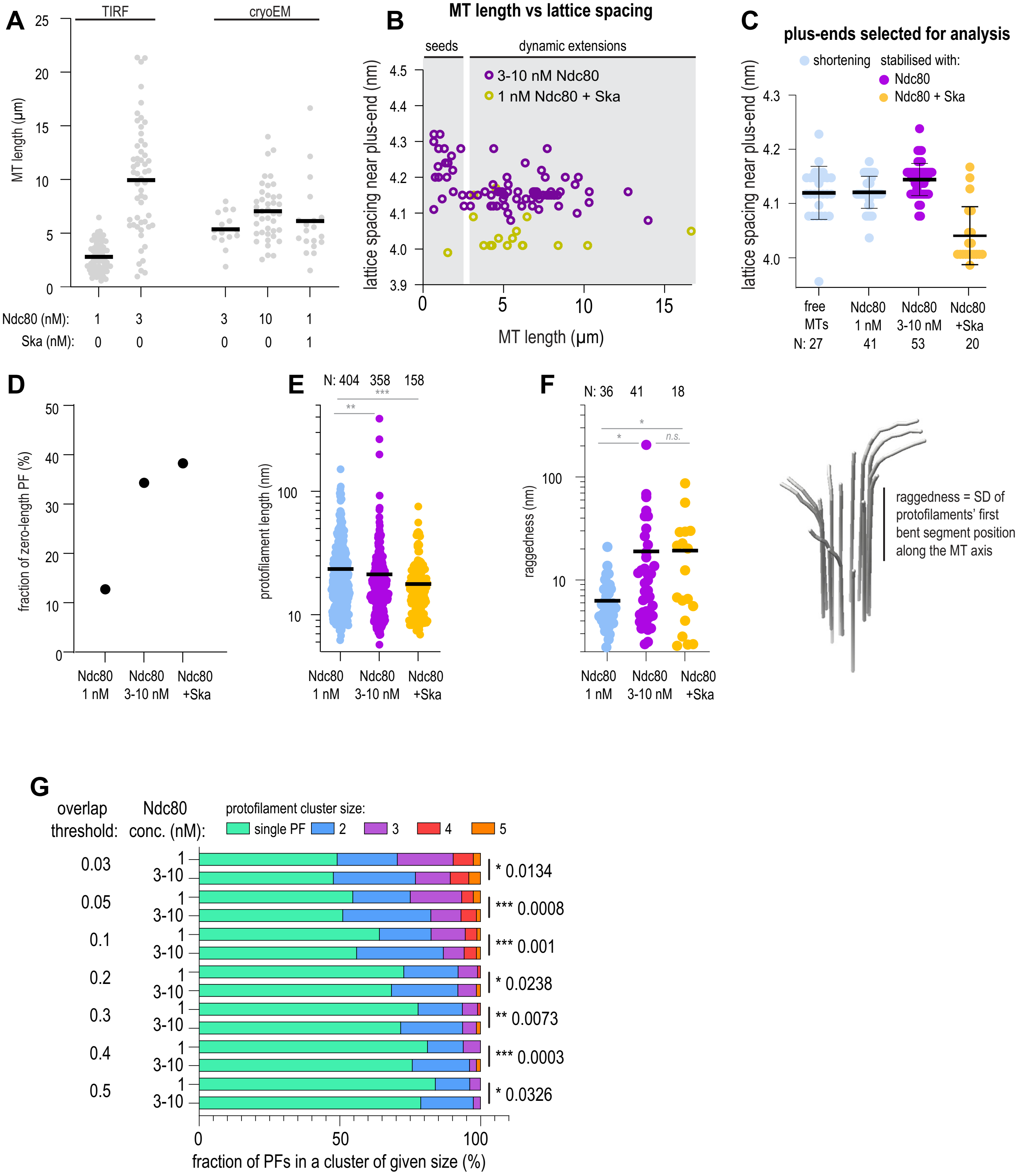
A
TIRF
cryoEM
MT length (µm)
25
20
15
10
5
0
Ndc80 (nM): 1 3 3 10 1
Ska (nM): 0 0 0 0 1
B
MT length vs lattice spacing
seeds
dynamic extensions
3-10 nM Ndc80
1 nM Ndc80 + Ska
lattice spacing near plus-end (nm)
4.5
4.4
4.3
4.2
4.1
4.0
3.9
0
5
10
15
MT length (µm)
C
plus-ends selected for analysis
shortening
stabilised with:
Ndc80
Ndc80 + Ska
lattice spacing near plus-end (nm)
4.3
4.2
4.1
4.0
free MTs
Ndc80 1 nM
Ndc80 3-10 nM
Ndc80 +Ska
N: 27 41 53 20
D
fraction of zero-length PF (%)
50
40
30
20
10
0
Ndc80 1 nM
Ndc80 3-10 nM
Ndc80 +Ska
E
N: 404 358 158
***
**
protofilament length (nm)
100
10
Ndc80 1 nM
Ndc80 3-10 nM
Ndc80 +Ska
F
N: 36 41 18
*
*
n.s.
raggedness (nm)
100
10
Ndc80 1 nM
Ndc80 3-10 nM
Ndc80 +Ska
raggedness = SD of protofilaments' first bent segment position along the MT axis
G
overlap threshold:
Ndc80 conc. (nM):
protofilament cluster size:
single PF
2
3
4
5
0.03
0.05
0.1
0.2
0.3
0.4
0.5
1
3-10
* 0.0134
*** 0.0008
*** 0.001
* 0.0238
** 0.0073
*** 0.0003
* 0.0326
0
50
100
fraction of PFs in a cluster of given size (%)

 © The Author(s)

Figure EV3. Criteria to select microtubule plus-ends for analysis, and effect of the protofilament overlap parameter on the observed differences if protofilament clustering.

(**A**) Microtubule length determined using TIRF microscopy, or low-magnification cryoEM in conditions used to detect target positions for tomography, in presence of Ndc80 and Ska in indicated concentrations. Grey circles: individual microtubules, lines: median. N: TIRF 1 nM Ndc80 (68), 3 nM Ndc80 (54); cryoEM 3 nM Ndc80 (15), 10 nM Ndc80 (41), 1 nM Ndc80 with 10 nM Ska (19). (**B**) Correlation of tubulin lattice spacing and microtubule length in the samples containing Ndc80 only (magenta), or Ndc80 + Ska (yellow). (**C**) Lattice spacing near plus-ends of microtubules selected for further analysis of protofilament shapes (mean ± SD). N: free MT (27), 1 nM Ndc80 (41), 3–10 nM Ndc80 (53), 1 nM Ndc80 with 10 nM Ska (20). (**D**) Fraction of protofilaments without a bent part in a microtubule plus-end. (**E**) Length of bent protofilament segments at microtubule plus-ends in presence of Ndc80 or Ndc80 + Ska. N: 1 nM Ndc80 (404), 3–10 nM Ndc80 (358), 1 nM Ndc80 with 10 nM Ska (158). Kolmogorov-Smirnov *p*-values: Ndc80 1 nM vs 3–10 nM: 0.0014 (**); Ndc80 3–10 nM vs Ndc80 + Ska: 0.4486 (n.s.); Ndc80 1 nM with vs without Ska: 0.0004 (***). (**F**) Raggedness of microtubule ends in conditions indicated. N: 1 nM Ndc80 (36), 3–10 nM Ndc80 (41), 1 nM Ndc80 with 10 nM Ska (18). Welch's t-test *p*-values: Ndc80 1 nM vs 3–10 nM: 0.0225 (*); Ndc80 3–10 nM vs Ndc80 + Ska: 0.9637 (n.s.); Ndc80 1 nM with vs without Ska: 0.0238 (*). (**G**) Protofilament cluster distributions obtained using indicated threshold value for neighbouring protofilament overlap. Chi-squared *p*-values: threshold of 0.03: 0.0134; threshold of 0.05: 0.0008; threshold of 0.1: 0.001; threshold of 0.2: 0.0238; threshold of 0.3: 0.0073; threshold of 0.4: 0.0003; threshold of 0.5: 0.0326.

© The Author(s)

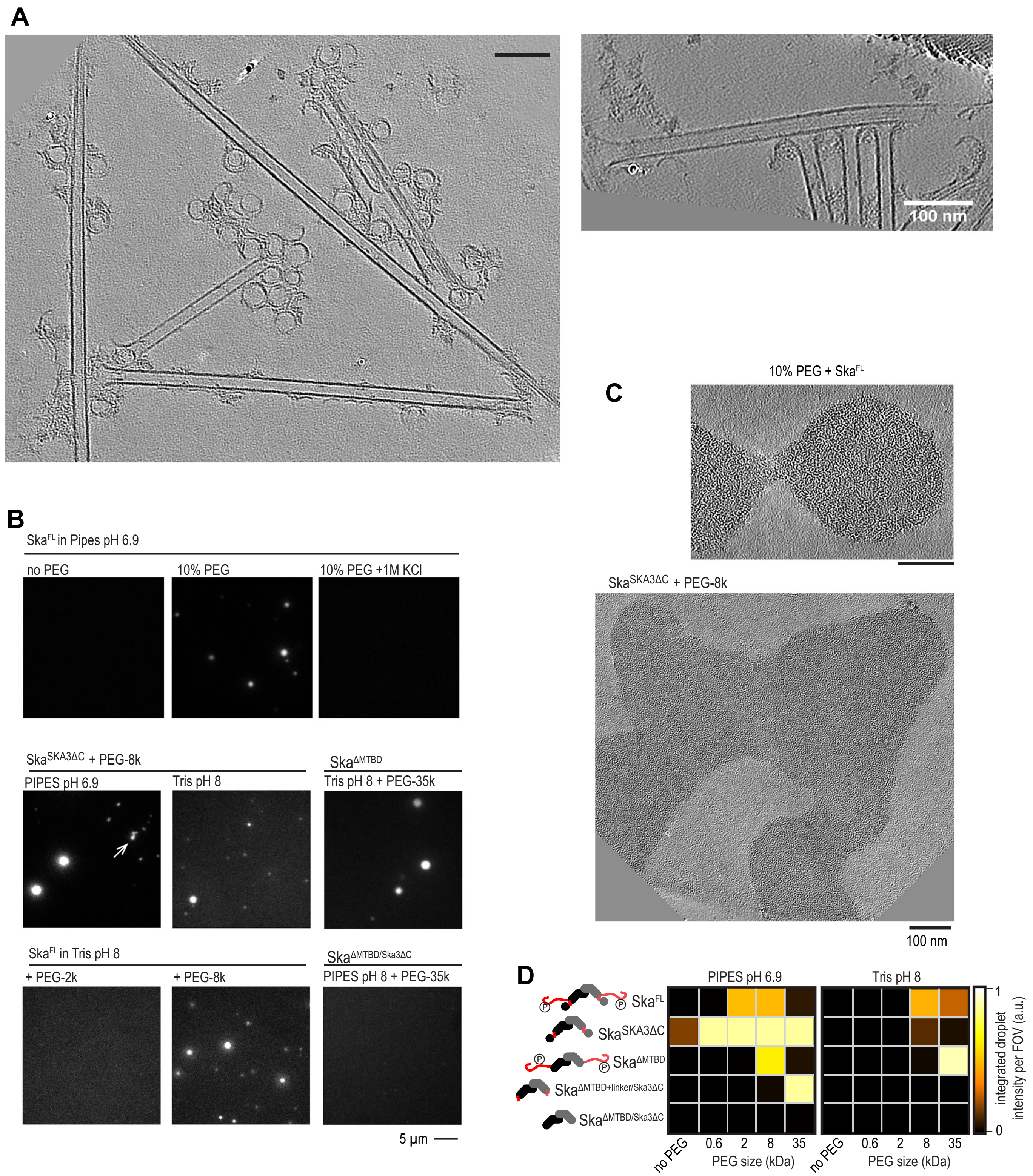

Figure EV4. Self-interaction of the Ska complex with and without microtubules.

(**A**) Representative slices through tomograms obtained in a sample containing Ska and dynamic microtubules. (**B**) Fluorescence microscopy images of Ska in presence or absence of crowding agents and various buffer compositions indicated on the panel. Arrow points to non-spherical aggregates of $Ska^{SKA3\Delta C}$. (**C**) Slices through tomograms of FL Ska or $Ska^{SKA3\Delta C}$ in presence of PEG. (**D**) Formation of fluorescent self-assembling oligomers of Ska in two various buffers, with the deletion construct indicated, and using a crowding agent indicated.

 © The Author(s)

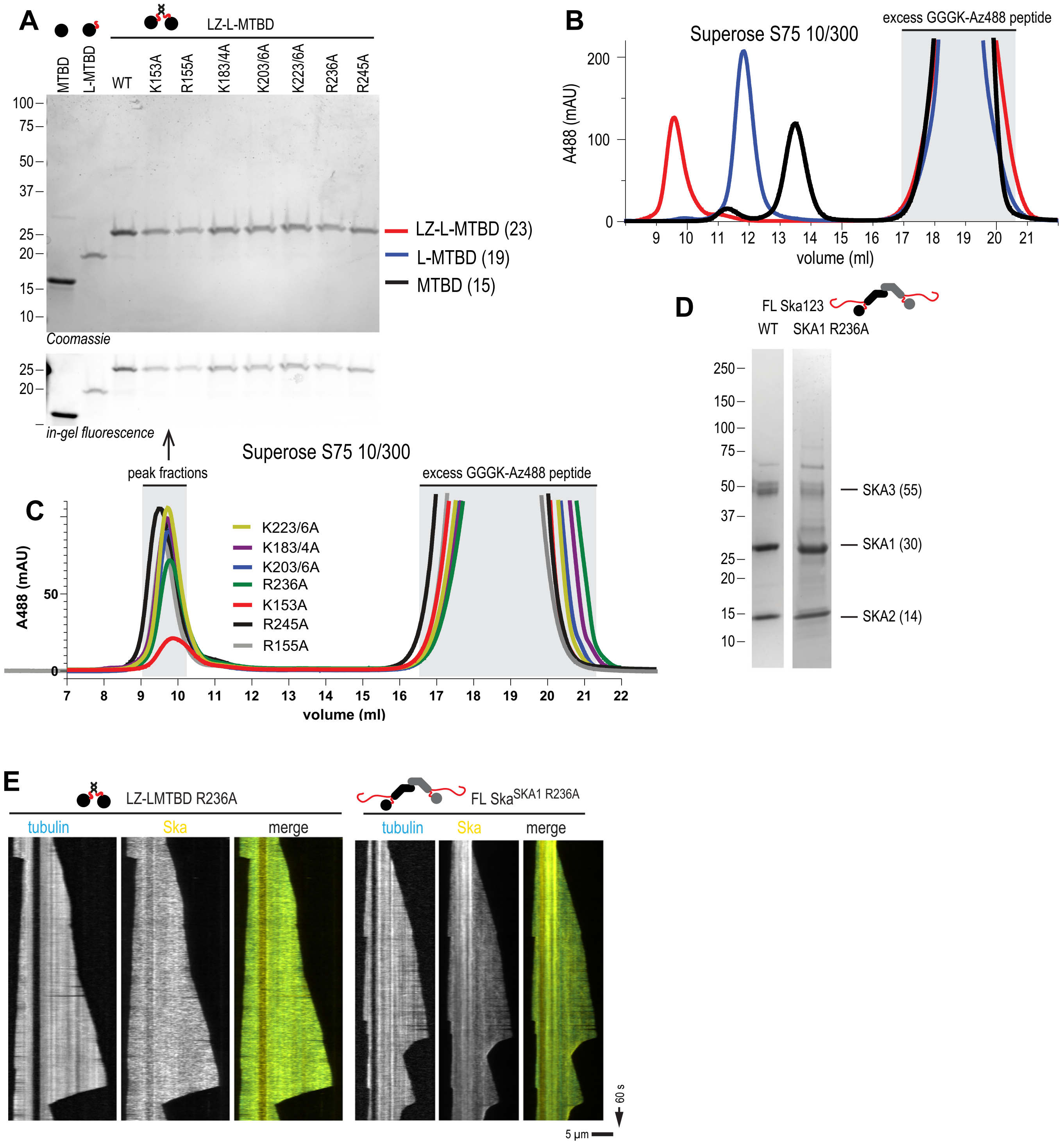

Figure EV5. Purification of SKA1 fragments, and additional kymographs demonstrating uniform microtubule coating by SKA1 R236A.

(**A**) SDS-PAGE of SKA1 MTBD constructs used in the study. (**B**) SEC profiles of dimeric LZ-LMTBD (red), and monomeric L-MTBD (blue) and MTBD (black) following fluorescent labelling with sortase. (**C**) SEC profiles of all point mutants of LZ-LMTBD following fluorescent labelling with sortase. (**D**) SDS-PAGE of FL Ska$^{SKA1\ wt}$ and FL Ska$^{SKA1\ R236A}$. (**E**) Additional examples of uniform coating of microtubules by SKA1 LZ-LMTBD R236A and FL Ska$^{SKA1\ R236A}$.

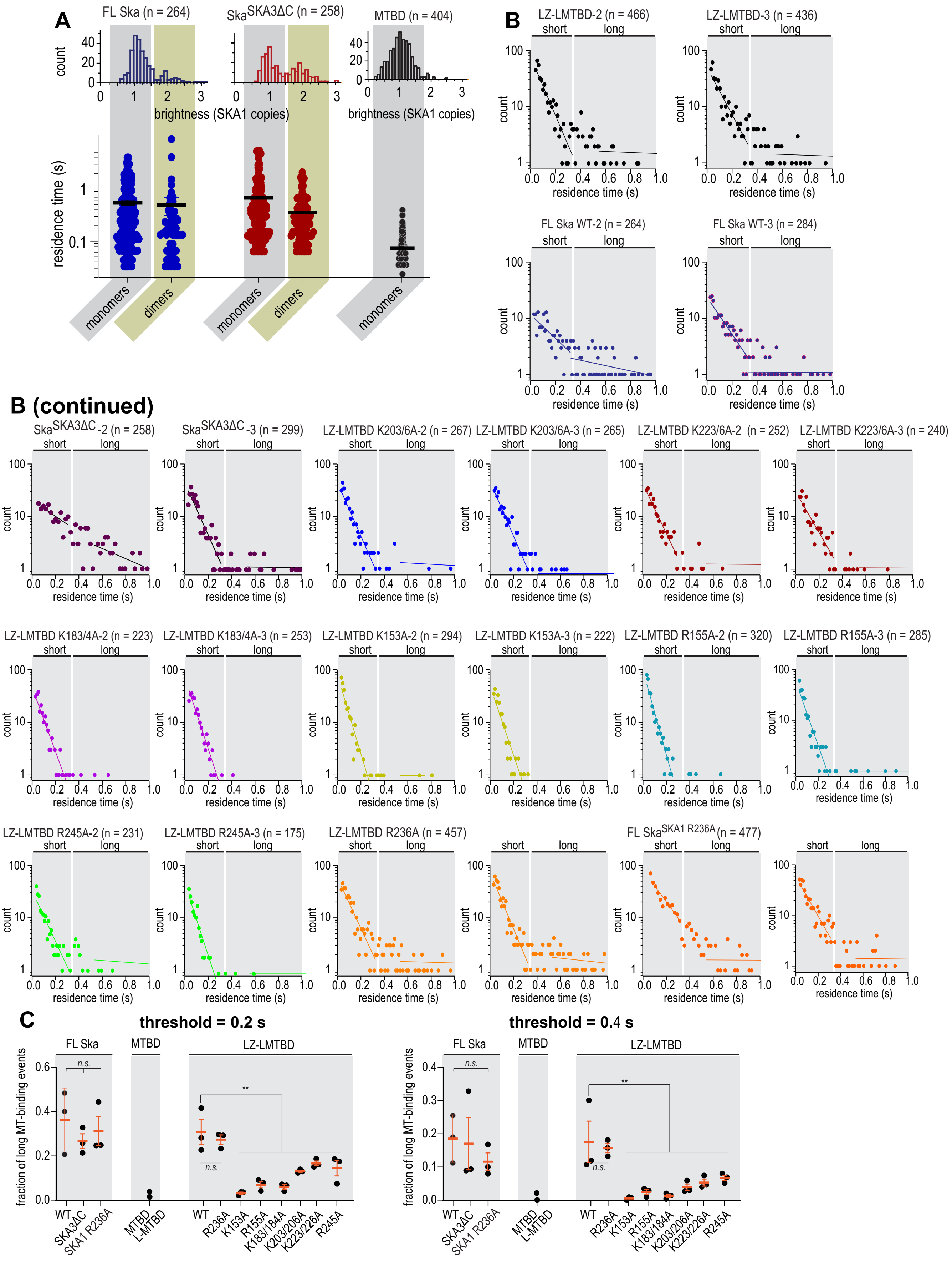
A
FL Ska (n = 264)
Ska$^{SKA3\Delta C}$ (n = 258)
MTBD (n = 404)
count
brightness (SKA1 copies)
residence time (s)
monomers
dimers
B
LZ-LMTBD-2 (n = 466)
LZ-LMTBD-3 (n = 436)
FL Ska WT-2 (n = 264)
FL Ska WT-3 (n = 284)
short
long
B (continued)
Ska$^{SKA3\Delta C}$-2 (n = 258)
Ska$^{SKA3\Delta C}$-3 (n = 299)
LZ-LMTBD K203/6A-2 (n = 267)
LZ-LMTBD K203/6A-3 (n = 265)
LZ-LMTBD K223/6A-2 (n = 252)
LZ-LMTBD K223/6A-3 (n = 240)
LZ-LMTBD K183/4A-2 (n = 223)
LZ-LMTBD K183/4A-3 (n = 253)
LZ-LMTBD K153A-2 (n = 294)
LZ-LMTBD K153A-3 (n = 222)
LZ-LMTBD R155A-2 (n = 320)
LZ-LMTBD R155A-3 (n = 285)
LZ-LMTBD R245A-2 (n = 231)
LZ-LMTBD R245A-3 (n = 175)
LZ-LMTBD R236A (n = 457)
FL Ska$^{SKA1\ R236A}$ (n = 477)
C
threshold = 0.2 s
threshold = 0.4 s
fraction of long MT-binding events
FL Ska
MTBD
LZ-LMTBD
n.s.
**
WT
SKA3ΔC
SKA1 R236A
MTBD
L-MTBD
R236A
K153A
R155A
K183/184A
K203/206A
K223/226A
R245A

 © The Author(s)

Figure EV6. Distributions of residence time of SKA mutants on stable microtubules.

(**A**) Residence times of FL Ska, $Ska^{SKA3\Delta C}$, and SKA1 MTBD molecules assigned into "monomers" and "dimers" based on their fluorescence intensity (circles), with mean and SEM (lines). FL Ska monomers ($n = 212$) vs dimers ($n = 52$): $p = 0.0988$. $Ska^{SKA3\Delta C}$ monomers ($n = 151$) vs dimers ($n = 102$): $p = 0.0098$. (**B**) Additional repeats of residence time measurements of constructs indicated. (**C**) Fraction of long microtubule-binding events of constructs indicated determined at two threshold values for the short/long boundary. Black dots: individual repeats, orange lines: mean and SD. Threshold = 0.2 s. Welch's t-test: FL $Ska^{SKA1\,wt}$ vs FL $Ska^{SKA1\,R236A}$ $p = 0.65$ (n.s.); LZ-LMTBD wt vs LZ-LMTBD R236A $p = 0.98$ (n.s.); FL vs LZ-LMTBD $p = 0.40$ (n.s.); FL $Ska^{SKA1\,R236A}$ vs LZ-LMTBD R236A $p = 0.62$ (n.s.); 1-way ANOVA of LZ-LMTBD wt vs non-R236A mutants $p = 0.0066$ (**). Threshold = 0.4 s. Welch's t-test: FL $Ska^{SKA1\,wt}$ vs FL $Ska^{SKA1\,R236A}$ $p = 0.24$ (n.s.); LZ-LMTBD wt vs LZ-LMTBD R236A $p = 0.98$ (n.s.); FL vs LZ-LMTBD $p = 0.48$ (n.s.); FL $Ska^{SKA1\,R236A}$ vs LZ-LMTBD R236A $p = 0.27$ (n.s.); 1-way ANOVA of LZ-LMTBD wt vs non-R236A mutants $p = 0.0069$ (**). Scale bar: 5 µm (horizontal), 1 s (vertical).

© The Author(s)

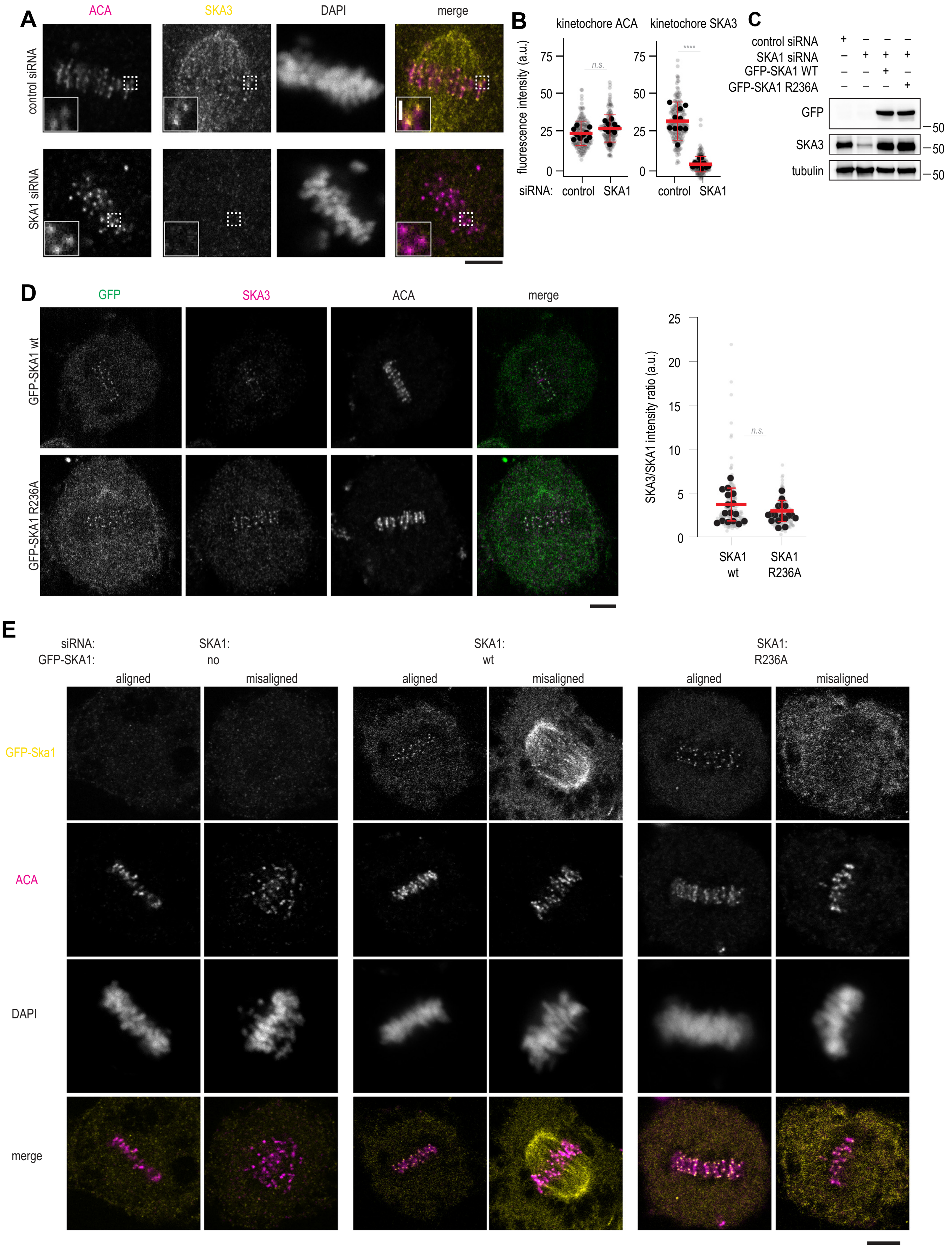
A
ACA
SKA3
DAPI
merge
control siRNA
SKA1 siRNA
B
kinetochore ACA
kinetochore SKA3
fluorescence intensity (a.u.)
75
50
25
0
n.s.
****
siRNA: control SKA1
control SKA1
C
control siRNA + – – –
SKA1 siRNA – + + +
GFP-SKA1 WT – – + –
GFP-SKA1 R236A – – – +
GFP
SKA3
tubulin
50
50
50
D
GFP
SKA3
ACA
merge
GFP-SKA1 wt
GFP-SKA1 R236A
SKA3/SKA1 intensity ratio (a.u.)
25
20
15
10
5
0
n.s.
SKA1 wt
SKA1 R236A
E
siRNA:
GFP-SKA1:
SKA1:
no
SKA1:
wt
SKA1:
R236A
aligned
misaligned
GFP-Ska1
ACA
DAPI
merge

 © The Author(s)

Figure EV7. Additional characterisation of cells depleted of SKA1 and expressing GFP-SKA1 with and without the R236A mutation.

(**A**) Single planes from z-stacks of confocal images of cells treated with control siRNA or SKA1 siRNA and stained for ACA (magenta), SKA3 (yellow), and DAPI. (**B**) Quantification of SKA3 fluorescence intensity at kinetochores following treatment with the indicated siRNA. N = at least 20 kinetochores per cell (small symbols), 10 cells per condition (large symbols). Welch's t-test *p* value: ACA (control vs SKA1 siRNA): 0.0652; SKA3 (control vs SKA1 siRNA): $1.2 \cdot 10^{-6}$ (****). Lines show mean and S.D. (**C**) Western blots probing for GFP, SKA3, and tubulin as a loading control, using cells treated according to conditions indicated at the top of the panel. (**D**) Single planes from z-stacks of confocal images of cells expressing GFP-SKA1 wt or R236A following SKA siRNA treatment, and stained for GFP (green) ACA (magenta), SKA3, and DAPI. The graph shows SKA3 intensity values normalised to SKA1 intensity per kinetochore (grey) or per cell (black). Red lines show mean and SD. N = 248 kinetochores, 16 cells (SKA1 wt); 266 kinetochore, 17 cells (SKA1 R236A). Welch's t-test: 0.1973. (**E**) Examples of cells considered having aligned or misaligned metaphase plates following the treatment indicated and stained for GFP-SKA1, ACA, and DAPI. Scale bars: 5 µm.

© The Author(s)
